# Can Engineered “Designer” T Cells Outsmart Chronic Hepatitis B?

**DOI:** 10.1155/2010/901216

**Published:** 2010-09-21

**Authors:** U. Protzer, H. Abken

**Affiliations:** ^1^Institut für Virologie, Technische Universität München/Helmholtz Zentrum München, 81675 München, Germany; ^2^Zentrum für Molekulare Medizin Köln und Klinik I für Innere Medizin Köln, Universität zu Köln, Robert-Koch-Str. 21, 50931 Köln, Germany

## Abstract

More than 350 million people worldwide are persistently infected with human heptatitis B virus (HBV) and at risk to develop liver cirrhosis and hepatocellular carcinoma making long-term treatment necessary. While a vaccine is available and new antiviral drugs are being developed, elimination of persistently infected cells is still a major issue. Recent efforts in adoptive cell therapy are experimentally exploring immunotherapeutic elimination of HBV-infected cells by means of a biological attack with genetically engineered “designer” T cells.

## 1. Persistent Hepatitis B Virus Infection in the Liver Is at Risk to Develop Cirrhosis and Hepatocellular Carcinoma

Human hepatitis B virus (HBV) is an enveloped, DNA virus with an icosaedral capsid, which belongs to the family of hepadnaviridae with a very narrow host range and a strong liver tropism. HBV has a 3.2 kb DNA genome in an extremely compact organisation containing four unidirectional overlapping open reading frames. Following infection the relaxed circular (rc) viral DNA is transported into the nucleus where it is filled up to form the so-called covalently closed circular DNA (cccDNA) in the nucleus which serves as template for transcription of the pregenomic, the precore and three subgenomic RNAs. The pre-genomic RNA is translated into the polymerase and the viral core protein and is encapsidated together with the HBV polymerase into the newly formd viral capsid. The pre-core RNA encodes the secretory HBeAg. Subgenomic RNAs are translated into the large (L), middle (M), and small (S) viral envelope proteins and the regulatory protein X. Infected cells secrete complete virions as well as subviral particles which represent empty envelopes packed with S protein. Detection of subviral particles in the serum as HBsAg can be used to identify HBV infected individuals (for review, see [1, 2]). 

While infection during the first year of life results in chronic, often life-long infection in >90% of individuals, infection during adulthood is mostly cleared resulting in life-long protective immunity. Clearance is accompanied by a vigorous, polyclonal HBV-specific T cell response. Persistent liver infection may cause substantial inflammatory liver disease associated with elevation of serum transaminases, necroinflammation, and tissue damage, which is classified as chronic hepatitis B. More than 25% of chronic hepatitis B patients develop progressive liver disease resulting in liver cirrhosis and/or hepatocellular carcinoma; 15%–40% of patients develop serious sequelae accounting for 0.6 to 1.2 million deaths worldwide per year. These figures demonstrate the urgent need in improving therapeutic strategies for the treatment of chronic hepatitis B.

## 2. Current Therapeutic Options in HBV Elimination Are Limited: A Rationale for a Cellular Immune Therapeutic Approach

Current therapeutic options in the treatment of chronic hepatitis B aim to suppress HBV replication and to induce remission of liver disease [3, 4] based on the fact that high viraemia is the major risk for progression of HBV-related liver disease and development of haptocellular carcinoma [5]. Standard treatment includes IFN-*α* or nucleoside or nucleotide analogues. IFN-*α* has direct anti-viral activity and moreover immune modulatory capacities, thereby stimulating the host immune response. Seroconversion of HBeAg positive patients to anti-HBe and loss of serum HBV DNA, however, occurs only in about 20% of treated patients and full immunological control of the virus indicated by loss of the HBsAg in maximally 5% [6]. In many cases, moreover, response to treatment is not durable. 

IFN-*α* therapy is a difficult treatment option for a number of applications and excludes therapy of advanced or decompensated liver disease due to severe side effects including hepatitis flares, fever, myalgias, thrombocytopenia, and psychic depression. Many inhibition of viral reverse transcriptase by nucleoside analogues impairs the viral life cycle but does not target the episomal replication template in the host cell allowing HBV cccDNA to persist. HBV cccDNA can persist even at low copy numbers in infected cells for months and years, being the initiating template for a new HBV replication cycle after the end of therapy which often results in disease relapse [7]. DNA containing capsids can replenish cccDNA in infected cells which makes a complete block in replication for a prolonged time span mandatory in order to eliminate cccDNA during cell division. Although the half-life time in humans is unknown, data from experimental systems imply a half-life of 9–14 days in chimpanzees and up to 50 days in duck and wookchuck models under therapy [8, 9]. Nucleoside analogues control HBV replication but usually do not eliminate the virus. Long-term, highly potent antiviral therapy using nucleotide analogue adefovir reduced virus replication and cccDNA levels; HBV cccDNA persists in the host cell, continuously produces HBsAg, and remains the potential source of viral rebound and disease recurrence after initial successful therapy. The overall number of HBV antigen positive cells, however, remains constant [10]. In patients who do not serocovert from HBsAg to anti-HBs, virus still persists in 5%–30% of hepatocytes indicated by HBsAg expression [11]. This situation makes long-term treatment necessary which, however, increases the risk for selecting resistant virus variants leading to hepatitis flares and hepatic decompensation [12]. Newly developed more potent nucleos(t)ide analogues avoid rapid development of therapy resistance. However, sustained immunological control of HBV infection, as substantiated by the loss of HBsAg and sustained loss of virus replication is so far very rarely achieved [13]. As a consequence, long-term or even life-long treatment is required and/or a persistent host anti-HBV immune response has to be established. This may be achieved by therapeutic vaccination to induce a specific antibody response or by adoptive transfer of HBV, specific T cells.

Vaccination is currently the most effective measure to reduce the global incidence of hepatitis B (for review, see [14]). Hepatitis B vaccination has been shown to preclude HBV infection effectively when used as a preexposure prophylaxis. Recombinant hepatitis B vaccines are well tolerated. Side effects are generally mild, transient, and confined to the site of injection. Although safety of the vaccine has been questioned in recent years severe side effects could not be demonstrated. In particular, some cases of relapse of multiple sclerosis or other demyelinating diseases were reported; the WHO Global Advisory Committee on Vaccine Safety concluded that the reported evidence does not support a link to hepatitis B vaccination. 

Following a full course of vaccination seroprotection in a preventive setting based on anti-HBsAg antibodies is close to 100% in children and almost 95% in healthy young adults and persists for at least 10 to 15 years once a good response has been achieved. In successfully immunized people clinically significant breakthrough infections are rare. To enhance immunogenicity and to confer more rapid and broader protection from hepatitis B, a new vaccine incorporating a novel adjuvant has recently been approved. Third-generation recombinant triple-antigen vaccines including pre-S1, pre-S2 and S antigen showed more effective for revaccination of people who are immune suppressed or had an inadequate response to current vaccines. Antibodies to the hepatitis B surface antigen are mainly targeted to bind the amino acid hydrophilic region, referred to as the “a” determinant of HBsAg, which provides protection against infection with all HBV genotypes and is responsible for the broad immunity afforded by hepatitis B vaccination. Viral variants with amino acid mutations in this region can escape antibody binding [15, 16] and hence infections in previously successfully vaccinated individuals. 

Taken together, vaccination remains the most effective means of preventing hepatitis B, liver cirrhosis and hepatocellular carcinoma. Compared to therapeutic interventions, vaccination is an economically attractive option, both in terms of cost-effectiveness and benefit-cost ratios. Once infection has established, however, vaccination with currently available vaccines is not effective.

The major limitation of vaccination is that it so far has no proven effect in HBV infected individuals. Adoptive transfer of cytotoxic T lymphocytes (CTLs) with specificity for HBsAg positive cells may represent an approach aiming to ultimately eliminate residual hepatocytes carrying HBV cccDNA. The rationale for adoptive T cell transfer in hepatitis B therapy is moreover given by the observation that a polyclonal CTL response with multiple specificities occurs in patients who had cleared acute infection [17, 18] whereas an oligoclonal and weak response is found in chronically infected individuals [19, 20]. While obviously capable in inducing viral clearance, CTLs are also thought to be also responsible for liver injury during HBV infection [21]. The latter may eventually cause hepatocellular carcinoma if the virus is not cleared and chronic inflammation persists. Two third HBV-infected leukaemia patients who occasionally received human progenitor cell transplants from HBV immune donors cleared their HBV infection [22]. Taken together, current data encourage the development of an adoptive T cell therapy to control not only virus replication but also disease progression. 

Adoptive T cell therapy recently showed substantial success in cancer immunotherapy after lymphodepletion [23–26] and demonstrated definite clinical benefit in relapsed leukaemia after allogeneic bone marrow transplantation and in Epstein-Barr virus-associated posttransplant lymphoproliferative disorders [27, 28]. Therapeutic efficacy in malignant diseases provides a strong rationale to develop adoptive T cell therapy also for chronic infectious diseases [18, 29]. Progress in the engineering of an artificial T cell receptor to deliver antibody like specificity in the context of a T cell activation signal [30] enables redirecting a cytolytic T cell response to predefined target cells as discussed below.

## 3. Adoptive Cell Therapy with Genetically Modified T Cells Specifically Redirects the Immune Response

Early attempts to engineer T cells with pre-defined specificity included the transgenic expression of a T cell receptor (TCR) resulting in genetically modified T cells which display the new specificity together with the endogenous TCR and which can be amplified to substantial numbers *ex vivo* before transplantation to the patient. Difficulties rose, however, in engrafting both recombinant TCR chains with sufficient efficacy. Lethal cytokine-driven autoimmune pathology can occur due to pairing of the introduced recombinant with the endogenous TCR chains in TCR gene-modified T cells [31]. This potential risk will not occur in the using of single chain chimeric receptors as outlined below. Most target cells, moreover, show defects in antigen processing and MHC presentation making them invisible for TCR recognition. This situation has led to the development of a chimeric, MHC-independent one-polypeptide receptor molecule whose antigen-binding domain is derived from an antibody and the signalling domain from the TCR. The archetypal chimeric antigen receptor (CAR; immunoreceptor), nicknamed “T-body”, consists of an extracellular single chain fragment of variable region antibody which is fused to the transmembrane and cytoplasmic domain providing signalling moieties, frequently the CD3*ζ* of the TCR. Due to its one-polypeptide chain design and modular composition, CARs substantially improved the technology in redirecting T cells by combining different advantages (Table 1). Upon antigen engagement of the extracellular domain, the signalling endodomain initiates T cell activation resulting in T cell amplification, secretion of proinflammatory cytokines, and cytolysis of those cells which express the targeted antigen on the cell surface. There are major differences between the TCR and a CAR since CARs are not MHC-restricted thus recognizing antigens not necessarily presented by the MHC. CAR redirected T cells can thereby target cells with established immune escape mechanisms like down MHC regulation or reduced endolysosomal antigen processing. An additional advantage with particular relevance for clinical applications is that a CAR recognises targets which are shared by many individuals independently of their MHC haplotype. 

Optimisation the individual CAR domains has been a long standing focus of research, in most cases with respect to deliver the appropriate T cell activation and to provide sustained survival signals (for details, we refer to recent reviews [30, 32]). The modular structure enabled further additions of signalling domains to the cytoplasmatic part, for example, costimulatory domains to endow T cells with full activation potential independent of the costimulatory ligand on target cells [33, 34]. CAR engineering together with efficient and safe technologies for gene transfer to T cells using *γ*-retroviral or lentiviral vectors have become the method of choice to redirect specificity of T cells for adoptive cell therapy. The technical procedures to engineer redirected T cells from patient peripheral blood T cells have been adapted to a GMP conform process [35, 36]. The majority of CARs were generated to target antigens (non-exclusively) expressed on cancer cells; the focus of research on CAR redirected immunotherapy is therefore dedicated to cancer therapy (we refer to recent reviews [28, 30, 32]). While CAR modified T cells for the elimination of cancer cells is currently evaluated in clinical trials, interest is raising to redirect cytotoxic T cells to persistently virus infected cells, cells of major interest are human immunodeficiency virus-1- (HIV-1-) infected T cells [37] and HBV-infected hepatocytes [34] displaying viral proteins on the cell surface.

## 4. Engineered T Cells with Specificity for HBV Infected Cells Provide a Novel Therapeutic Option

HBV-infected cells continuously produce HBsAg which is mainly composed out of the HBV S with trace amounts of M and large L surface proteins [38]. The HBV antigens are present on the surface of HBV replicating cells [39], based on the fact that HBV surface proteins are incorporated into the ER membrane which is in steady exchange with the plasma membrane. The proteins are, moreover, a sensitive and specific marker for active viral replication in HBV infected cells. These proteins thereby represent potential targets for an adoptive cell therapy using redirected cytotoxic lymphocytes. Recently, two CARs directed against two different HBV surface antigens, L- and S-protein, were described to redirect a cytolytic T cell response to HBV infected hepatocytes [40]. Upon S-protein engagement and to a lesser extent by targeting L-antigen, CAR engineered T cells were activated to secrete proinflammatory cytokines, to proliferate and to eliminate HBV-replicating hepatoma cells as well as HBV infected primary human hepatocytes *in vitro*. 

Since the choice of the CAR-binding domain is of particular relevance for the clinical success of an adoptive T cell therapy, an scFv antibody was used for targeting which is directed against a conformational epitope in the “a” determinant of S-protein [41]. This antibody showed highest binding efficiency and specificity and proved most effective and specific in CAR redirected T cell activation. To obtain a suitable scFv against L-protein, which is only in trace amounts present in subviral particles, a scFv antibody was derived from the well-established monoclonal antibody 5a19. Antigen recognition by CAR engineered T cells resulted in high-level IFN-*γ* secretion *in vitro*. Secreted IFN-*γ* is assumed to control HBV replication thereby itself exhibiting an antiviral effect [42, 43]. By combining the CD3*ζ* signalling domain with the CD28 costimulatory moiety in a so-called “second generation” CAR IL-2 secretion is additionally induced providing full T cell activation. Although the targeted antigens are expressed on different levels both anti-S and anti-L CAR engineered T cells lysed with similar efficiencies HBV-replicating hepatoma cells and HBV infected primary human hepatocytes. However, cytokine levels, and particularly IL-2 levels, were markedly higher when T cells grafted with anti-S CAR were used in comparison to anti-L CAR-engineered T cells. This difference is likely to be due to the high abundance of the S-protein expressed and subsequently displayed on the surface of HBV infected cells and is reflected by time course experiments which showed accelerated activation kinetics of S-specific CAR in comparison to L-specific T cells. 

CAR engineered T-cells bind not only HBV-infected cells but also soluble HBV particles leading to NF-*κ*B activation and IFN-*γ* secretion indicating that the surface area of HBV viral and subviral particles is sufficient to induce CAR clustering. This is in contrast to soluble monomeric proteins like CEA which does not induce CAR-mediated T cell activation due to lack of CAR clustering [44]. Anti-HIV-1 gp120 CAR engineered T cells, for comparison, are less efficiently activated by the monomeric soluble gp120 than by binding to the transmembrane protein of infected cells [37]. This does, however, neither lead to killing of infected hepatocytes incubated with viral and subrial particles nor impair cytolytic activity of anti-HBV CAR T cells *in vitro* by soluble HBV particles in HBsAg levels of up to 250 ng/mL, relating to approximately 40,000 particles per T cell [32]. 

Using primary human hepatocytes infected with HBV as targets and primary T cells from the same donor for engineering with anti-S and anti-L CAR, engrafted T cells were activated for hepatocyte killing. About 10–20% hepatocytes were *in vitro* infected, engineered T cells specifically killed a subfraction of hepatocytes. HBV-infected hepatocytes were eliminated since >95% HBV cccDNA was eliminated from HBV infected hepatocyte cultures by anti-S CAR and >80% by anti-L CAR engineered T cells [40]. This strongly argues for specific elimination of HBV-infected hepatocytes [9, 45]. The conclusion is, moreover, sustained by the observation that levels of total intracellular HBV core protein were reduced by 73% by anti-S and by 57% by anti-L CAR T cells, levels of intracellular HBV rcDNA by 82% and 72%, respectively, whereas intracellular albumin indicating viability of hepatocyte cell population was only reduced by 17 and 14%, respectively. The data do not exclude, however, that activation of CAR redirected T cells could be accompanied to some extend by unspecific killing of uninfected hepatocytes in the neighbourhood of infected cells, so-called bystander killing.

CAR redirected T cells lyse their targets predominantly via granzyme/perforin. Accordingly, Lamp-2 staining on the surface of anti-S CAR T cells indicated degranulation of lytic granules upon contact to target cells. Additionally activation of effector caspase-3 and -7 were detected in targeted hepatocytes and cytotoxicity was blocked by a pan-caspase inhibitor. We, therefore, expected induction of caspase activated DNases which will degrade histone-bound nuclear cccDNA. HBV rcDNA which is encapsidated in viral capsids, however, was protected by the core protein and not degraded in consequence of a cytolytic T cell attack of infected cells.

Beside CTL mediated cytolysis of infected target cells, noncytolytic processes may equally contribute to the reduction of HBV cccDNA. The assumption is based on the observations that no massive hepatocyte lysis occurs during recovery of chimpanzees from HBV infection [46] and the number of CD8^+^ T cells infiltrating the liver does not correlate with the level of hepatocyte lysis [47]. CAR engineered T cells secrete proinflammatory, anti-viral cytokines, in particular type-1 interferon and TNF-*α*, which potentially can suppress viral replication through non-cytolytic, immune-mediated mechanisms which also contribute to diminish the cccDNA reservoirs from infected cells [46] because they kill infected cells and induce divison of hepatocytes which then are prone to loose cccDNA [48]. In the absence of cell division and in the chronic phase of the disease, these processes may be ineffective and cccDNA will persist with the long half-life of hepatocytes. 

HBV-infection may also be controlled by an innate response mediated by Kupffer cells and dendritic cells and involving different pathways [46, 49, 50]. This is sustained by the observation that chronic hepatitis B infection is associated with reduction of dendritic cell functions and impairment of the innate immune response [51], although it does not infect immune cells [52]. Toll-like receptor signalling is downregulated in the liver and blood of HBeAg-positive individuals [53, 54] which points to potentially crucial interactions between virions, HBeAg and the innate immune response. The relative contribution of cytolytic and non-cytolytic processes in the elimination of HPV cccDNA, however, is elusive yet. 

Taken together, two CARs with specificity for two different HBV surface proteins are well characterized to date. Upon retroviral expression, CARs deliver HBV specificity to primary human T cells, trigger their activation to secrete proinflammatory cytokines, and enable them to eliminate HBV infected cells from a primary human hepatocyte culture. Specific antigen recognition results in proliferative expansion of redirected T cells which is a key requisite for effective repopulation after adoptive transfer into patients. Soluble HB viral particles, however, bind to the CAR-modified T cells inducing some activation, at least *in vitro*, but do not block redirected T cell activation upon binding to HBV-infected target cells.

## 5. CAR-Engineered T Cells Redirected Against HIV-1-Infected Cells

Immunotherapy utilizing engineered T cells with specificity for virally infected cells has been studied in detail in the setting of HIV-1 infection. A CAR with a binding domain derived from a neutralizing anti-gp120 antibody redirects T cells towards HIV envelope glycoprotein gp120 resulting in an effector T cell response including cytokine secretion. Engineered CD8^+^ T cells induced lysis of HIV-1- infected CD4^+^ T cells with high efficiencies *in vitro *[37]. The strategy was, moreover, adapted to the particular situation of HIV infection by engineering a CD4-*ζ* CAR with the extracellular domain of CD4 which targets HIV env expressed on the surface of infected cells [54]. The MHC independency of such interaction allows HIV-specific targeting of both CD4^+^ and CD8^+^ T cells and may, moreover, circumvent the ability of HIV to evade T cell recognition through downregulation of HLA molecules on the surface of infected cells. CD4-*ζ* CAR modified cytotoxic T cells showed antigen-driven proliferation, cytokine release and cytolytic activity towards HIV-infected T cells in preclinical studies [55, 56]. Adoptive transfer of *ex vivo* amplified, syngeneic, CD4-*ζ* CAR modified CD8^+^ T cells in HIV-infected twin pairs, however, revealed a rapid decline in modified T cells in the peripheral blood [57]. This may be due to the lack of CD28 costimulation and the lack of help by CD4^+^ T cells, highlighting the need of CD28-*ζ* CARs and the *ex vivo* modification of both CD4^+^ and CD8^+^ T cell populations. In a phase II trial coinfusion of autologous CD4-*ζ*, CAR-modified CD4^+^ and CD8^+^ T cells with or without IL-2 supplementation in HIV-infected patients with detectable virus load resulted in persistence of modified T cells in long-term [58]. Taken together, CD4-*ζ* modified T cell can inhibit HIV replication, kill HIV-infected cells *in vitro*, and survive for prolonged periods *in vivo*. Infusion of CAR engineered, but not unmodified, T cells resulted in decrease in HIV burden in at least two assays and a trend toward fewer patients with recurrent viremia [59]. Given the rapidity of the HIV-1 life cycle, timing of cell lysis in the control of viral replication appears to be crucial since recognition by anti-gp120 CAR-modified T cells requires that infected cells express assembled viral envelope on the surface of target cells.

## 6. Translation to Clinical Use: Potency and Unresolved Questions

**Figure 1:**
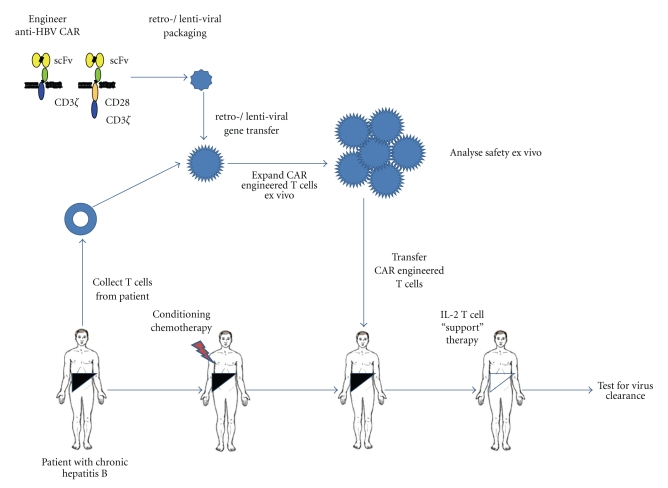
*Adoptive cell therapy using CAR engineered T cells.* T cells from the peripheral blood of a patient with chronic hepatitis B is obtained, *ex vivo* stimulated to proliferate, engineered with HBV specific CAR by retro- or lentiviral gene transfer, amplified to therapeutic numbers and readministered by i.v. infusion to the patient along with low dose IL-2. To allow homeostatic expansion of adoptively transferred T cells, patient may be pretreated by nonmyeloablative lymphodepletion with cyclophosphamide and fludarabine. T cell application may be performed repetitively using the same batch of *ex vivo* CAR engineered T cells.

CAR-engineered T cells have been applied in phase-I clinical trials, mostly in the treatment of malignant diseases, and several trials are ongoing or in advanced phase of preparation (for review, see [28]). All trials have in common *ex vivo* engineering of patient peripheral blood T cells with the recombinant CAR, extensive *ex vivo* amplification and reinfusion of those cells, mostly by repetitive applications, into a patient who is pretreated for lymphoablation to allow homeostatic expansion of engineered T cells (Figure 1). Preclinical studies and clinical trials have been designed based on lessons learned from the preclinical animal models, for example, the use of a humanized scFv for CAR binding, inclusion of the CD28 costimulatory domain to sustain T cell survival, use of *γ*-retro- or lentiviral vectors or electroporation for T cell modification, inclusion of homeostatic interleukins to amplify engineered T cells, the use of both CD4^+^ and CD8^+^ T cells or the use of autologous T cells specific to viral antigens such as EBV or influenza (for review, see [32]). A proof-of-concept clinical trial in which patients with relapsed or refractory indolent B cell lymphoma or mantle cell lymphoma were treated with autologous, anti-CD20 CAR modified T cells was reported the Fred Hutchinson Cancer Research Center [26]. Patients received a total of 20 T cell infusions with minimal toxicities. Modified T cells persisted *in vivo* up to 9 weeks in patients who additionally received low-dose subcutaneous IL-2 injections. Of the 7 treated patients, 2 maintained a previous complete response, 1 achieved a partial response, and 4 had stable disease. Another recent clinical trial by the Brenner group [60] has shown a correlation between the persistence of the adoptive transferred T bodies and the clinical outcome. While a number of trials in cancer treatment have recently been reported, adoptive T cell therapy aiming to eliminate HBV infected cells has not been performed so far. 

The two obvious advantages of the CAR strategy to eliminate HBV-infected cells by adoptive T cell therapy are the production of nearly unlimited numbers of immune cells with defined specificity and the MHC-independent targeting of virally encoded cell surface proteins (Table 1). Virus infected cells frequently downregulate proteins associated with antigen processing and presentation including MHC, which renders the infected cell invisible to T cells. A MHC-independent T cell attack redirected by an antibody derived CAR may be of significant advantage in this situation. While the CAR structure plays key roles in optimizing the function of engineered T cells, critical to the success of therapy will be the choice of targeted antigen. By using antibodies for binding, a variety of viral antigen epitopes can potentially be targeted, each of which needs to be considered with respect to several parameters including the accessibility for the antibody and the capacity to cluster the CAR on the T cell surface. The spatial relationship of the targeted epitope with respect to the membrane topology of the target cell has to be taken into account since an optimal T cell-to-target cell spacing distance seems to be required for the most effective T cell response. Phage display and other powerful selection systems are assumed to provide a plethora of binding domains to target various epitopes of HBV encoded surface proteins. The affinity of a given scFv can moreover be increased or reduced [61, 62] in order to improve specificity and optimize T cell activation. Increase in affinity, however, does not necessarily result in increased activation of redirected T cells [61]. 

 The affinity dependent cross-reactivity to homologous antigens physiologically expressed on healthy tissues as well as potential inactivation of CARs by soluble antigens have to be taken into account. This may counterbalance advantages of increasing affinity and needs careful consideration and as such, empirical testing of the respective CARs.

Understanding of the mechanisms of CAR mediated killing of target cells is still at an early stage. *In vitro* analyses indicate that CAR redirected killing occurs predominantly via perforin/granzyme and less via Fas/FasL [63]. It is most likely that CAR-engineered T cells can proliferate and kill multiple targets, thereby amplifying the anti-viral response. 

Trafficking of T cells to the site of infection will determine the clinical success of adoptive T cell therapy. Engineered T cells are likely to pass the liver during first round of circulation. Whether they will infiltrate the tissue and kill infected hepatocytes needs to be proven in animal studies. In targeting HBV-infected hepatocytes, however, a number of questions still remain to be solved (Table 2). Does strong binding to individual HBV-infected cells trap the T cells locally? Will the immune suppressive microenvironment of the liver silence transferred T cells? What effect would circulating HBsAg or subviral HBV particles have on trafficking and activation of engineered T cells taking into account that the surface of HBV particles seems sufficient to induce CAR-mediated T cell activation?

A beneficial effector cell-to-target cell ratio at the site of HBV infected liver cells is likely to be required for efficient target cell elimination. An estimation based on clinical data, however, is not yet available from trials performed in tumor patients; however, higher numbers of engineered T cells, that is, 10^9^–10^10^ cells, per dose as well as multiple applications are likely to increase efficacy. Antigen-driven amplification of engineered T cells, on the other hand, may help to achieve a sufficient T cell response but may also be accompanied by a flare of hepatitis. Moreover, autoimmunity may potentially result from “off-target” T cell activation and may lead to targeting of healthy tissues. In case of virally encoded antigens, cross-reactivity with physiologically expressed human antigens is unlikely as long as scFv mediated CAR activation is specific. Autoimmunity has been a major problem in CAR targeting of carboanhydrase IX (G250), a renal cell carcinoma antigen also expressed at low levels on bile duct epithelia [24] and in a recent trial targeting Her2/neu (ErbB2) with fatal outcome [63]. In the renal cell carcinoma trial, toxicity was controlled using steroids to deplete modified T cells. Since virally encoded proteins are mostly genuine, the choice of targeted antigen is less critical than targeting tumor associated antigens which most frequently are physiologically expressed at lower levels in healthy tissues. Control procedures to ensure that off-target toxicities are kept to a minimum, however, need careful evaluation. Despite thoroughly pre-clinical safety testing, controlling the engineered T cell *in vivo* performance represents a mandatory option. Novel gene suicide systems using tagged CAR molecules which can be targeted by T cell depleting antibodies *in vivo* [64] and the induction of endogenous apoptosis pathways [65] have recently been developed to permit specific depletion of the engineered T cells rather than the need for total T cell depletion strategies.

Taken together, strong arguments support the development of this form of adoptive cell therapy for the treatment of persistent viral infections. Since the initial descriptions of the approach nearly 20 years ago, most of the early technological hurdles concerning the generation of antigen-specific T cells have been overcome. Insight is rising how effective CAR redirected T cells are when compared to “natural T cells” upon adoptive transfer *in vivo*. While first clinical trials show the safety, feasibility, and potential therapeutic activity of adoptive T-cell therapies using this approach, concerns raised that autoimmunity due to cross-reactivity with healthy tissues is a major safety issue. Introduction of genes into T cells using retroviral vectors has been proved safe. It is clear today that the risk of leukaemia that occurred in patients receiving retroviral vector-mediated gene transfer into hematopoietic stem cells does not exist for mature T cells [66]. The antibody whose scFv will serve to redirect the engineered T cells has to be carefully tested both in terms of specificity and affinity to diminish the risk of damaging essential healthy tissues. Despite a number of unresolved questions, engineered T cells redirected by a HBV S or L antigen specific CAR, potentially combined with ablation of suppressor cells, may provide an attractive strategy to eliminate HBV-infected cells by implementation of an effective virus antigen-specific CTL response. Animal experiments and subsequent clinical trials need to determine whether CAR redirected T cell activation can establish full immunological control of HBV infection.

## Figures and Tables

**Table 1:** Advantages and disadvantages of CAR redirected T cell therapy.

Advantages	Disadvantages
MHC-independent antigen targeting	empirical testing of newly generated CARs required
High affinity binding	optimal T cell-to-target cell spacing distance required for T cell activation
Allows production of high numbers of antigen-specific, patient-derived T cells	individual engineering of patient's T cells necessary
allows redirecting CD4^+^ and CD8^+^ T cells	short-term persistence of engineered T cells *in vivo *
redirection towards a broad variety of targets possible	T cell expansion may reduce reactivity
T cell expansion upon antigen engagement	antigen cross-reactivity increases risk of “off-target” activation and autoimmunity
Repetitive killing possible	soluble antigen may block T cell activation

**Table 2:** Open questions concerning the clinical use of CAR-engineered T cells in the treatment of chronic hepatitis B.

(i) Does binding to individual HBV infected cells trap the anti-HBV CAR T cells locally in the liver?	
(ii) To which extend are HBV-infected hepatocytes eliminated by engineered T cells?	
(iii) Will the immune suppressive microenvironment silence transferred T cells?	
(iv) What effect would circulating HBsAg or subviral HBV particles have on the activation of engineered T cells? Do soluble HBV particles	
induce CAR mediated “on-target off-organ” T cell activation?	
(v) Is HBV-specific T cell memory induced?	
(vi) How can induction of anergy of engineered T cells be prevented?	
(vii) Do inflammatory cytokines secreted by activated T cells attract a second wave of nonspecific inflammatory cells, and how do these	
cells comodulate the anti-HBV T cell response?	

## Abbreviations

CAR: Chimeric antigen receptor,
CTL: Cytotoxic T-lymphocyte,
GMP: Good manufacturing practise,
HBV: Hepatitis B virus,
HBsAg: Hepatitis B surface antigen,
cccDNA: Covalently closed circular DNA,
rcDNA: Relaxed circular DNA,
IFN: Interferon.
